# The Anatomy of Action Systems: Task Differentiation When Learning an EMG Controlled Game

**DOI:** 10.3389/fpsyg.2016.01945

**Published:** 2016-12-15

**Authors:** Ludger van Dijk, Anniek Heerschop, Corry K. van der Sluis, Raoul M. Bongers

**Affiliations:** ^1^Center for Human Movement Sciences, University of Groningen – University Medical Center GroningenGroningen, Netherlands; ^2^Department of Rehabilitation Medicine, University of Groningen – University Medical Center GroningenGroningen, Netherlands

**Keywords:** action systems, electromyography, motor learning, myoelectric control, serious gaming, transfer of training, games, perception-action

## Abstract

This study aims to determine to what extent the task for an action system in its initial development relies on functional and anatomical components. Fifty-two able-bodied participants were randomly assigned to one of three experimental groups or to a control group. As a pre- and post-test all groups performed a computer game with the same goal and using the same musculature. One experimental group also trained to perform this test, while the other two experimental groups learned to perform a game that differed either in its goal or in the musculature used. The observed change in accuracy indicated that retaining the goal of the task or the musculature used equally increased transfer performance relative to controls. Conversely, changing either the goal or the musculature equally decreased transfer relative to training the test. These results suggest that in the initial development of an action system, the task to which the system pertains is not specified solely by either the goal of the task or the anatomical structures involved. It is suggested that functional specificity and anatomical dependence might equally be outcomes of continuously differentiating activity.

## Introduction

When learning to perform a task, not only the means to achieve the goal of the task need to be learned, but the goal itself also refines as the action becomes more dexterous. For instance, when learning to play tennis, we at first only have a general idea of how to hit the ball. Over learning, however, we learn to discern the many ways a ball can approach, and develop different strokes to accommodate for this. Moreover, we learn to return the ball strategically, for example steering the opponent to the right of the court, and thus the goal of the stroke changes as well. In other words, during learning a reciprocal differentiation of both action and goal results in changes in the details of what constitutes the task over time. Within the theory of action systems (Reed, 1982, 1988, 1996), this reciprocal differentiation of both action and goal during learning has received comparatively little attention. Rather, the focus has been on fully differentiated systems.

According to Edward Reed’s theory of action systems, when acting, the human body is organized in a goal-directed way in order to attain a task (Reed, 1982; see also Gibson, 1979; Bernstein, 1996; Reed, 1996; Newell and Vaillancourt, 2001; Warren, 2006). The coordinated system that is reliably formed as a task is performed, called an “action system,” is characterized as being *functional* – that is, as being adapted to attain a certain goal in the environment. By looking at the effect that performing one task has on the performance of a subsequent, different, task (i.e., a transfer effect), research has shown that fully differentiated action systems are task-specific: they are strongly dependent on the availability of task-relevant information for their formation, but largely independent of the anatomical components taken up (see Rieser et al., 1995; Withagen and Michaels, 2002; Bruggeman and Warren, 2010; see also Kelso and Zanone, 2002; Seifert et al., 2013). Critically, however, the findings of anatomical independence assume that the task for the action system has been fully established.

Consequently, the theory of action systems, that takes the functional organization of action as its starting point has come to be taken to be at odds with studies that show the importance of anatomical components for action (e.g., Durgin et al., 2003; see also Bingham et al., 2014; Van Dijk et al., 2016a). By taking the learning of action systems into account, this paper aims to show that such an opposition is not implied. In particular, in this article we aim to look at the early learning of an action system in order to determine what constitutes the task for an action system in its early development. To make a start on this, we will first introduce the processes of calibration and exploration to show how in these processes both functional and anatomical aspects are always implicated while forming of an action system.

### Two Processes for Learning

A primary process in getting an action system to be functionally specific to a task is the process of “calibration.” This process maps the action system to the perceptual information necessary to perform a specific task (see Rieser et al., 1995; Withagen and Michaels, 2005; De Vries et al., 2015). In a seminal study for example, Rieser et al. (1995) showed that as long as information for forward movement (optic flow) is available to calibrate to, an action system for locomotion can be set up irrespective of the anatomy involved. In a transfer task, the specific mapping of locomotion to optic flow during walking influenced locomotion during side-stepping, but not to throwing or turning in place (see also Withagen and Michaels, 2002; Bruggeman and Warren, 2010). Calibration to perceptual information is thus independent of the anatomical components used, but instead relies on the availability of task-relevant information, such as the optic flow that specifies moving forward. This is in agreement with Kelso and Zanone (2002) who, starting from a slightly different theoretical paradigm, also showed that upon learning *task-specific* coordination dynamics (in their case learning relative phase movements of either the arms or the legs), visuomotor performance will improve across effector-systems (Kelso and Zanone, 2002). In other words, they showed such transfer is independent of the anatomical components figuring in the task.

Nonetheless, some studies have shown that the anatomical components taken up in the system can influence task performance (e.g., Durgin et al., 2003; Bingham et al., 2014). This has prompted Bingham et al. (2014) to refine the relationship between task-function and anatomical aspects in an important way. The study created a different discrepancy for each arm between the haptic and visual feedback for the location of an object to be reached (Bingham et al., 2014). While the visual feedback remained the same for each arm, haptically the object was either moved forward or backward – requiring the relation between perception and action to be re-calibrated for each arm independently.

In a transfer test, Bingham et al. (2014) showed that the resulting perception-action relationship did not transfer between arms. The study thus showed that to keep an action system adapted to its environment, if both limbs require a different perception-action relation, then they are functionally distinguished. In other words, discerning anatomical aspects can be the outcome of a functional process. Therefore, Bingham et al. (2014) proposed the “mapping theory of calibration.” They proposed that when adaptation to a task requires one limb to be mapped (i.e., calibrated) differently to the available information than the other limb, the two limbs get *functionally* differentiated based on the available feedback – in effect differentiating into two different tasks and thus into two separate action systems. In short, the task can come to include anatomical terms. As the process of calibration keeps an action system adapted to perform a certain task, it in turn too allows anatomical constraints to emerge as functionally relevant to task performance.

So anatomical aspects can emerge as task-relevant distinctions. However, the converse was also recently shown: task-relevant distinctions emerge on the basis of anatomical constraints. De Vries et al. (2015) showed that in an unfamiliar task in which the length of a stick needed to be estimated using either hands or feet the “education of attention,” that is the moving toward the most useful perceptual information (Jacobs and Michaels, 2007; De Vries et al., 2015), was partly constrained by the anatomy used. Crucially, the results of their experiment suggested that this was so because the ability to distinguish perceptual information with either hands or feet differed. In other words, the ability to *explore* for more useful information was constrained by the anatomical components taken up during performance (De Vries et al., 2015). Some anatomical aspects, it seemed, could not (yet) generate the appropriate type of information for acting. This implies that when the goal of the task is still unclear and the learning process is dominated by exploration for, rather than calibration to, information, the task might be partly distinguished by the anatomical components used.

Taken together, these studies suggest that, in principle, the theory of action systems covers the possibility of accounting for anatomical dependence. Although action systems are defined relative to a task and often end up as largely independent of their specific anatomical components, the task itself might be differentiated by the learner based, in part, on the anatomical constraints it faces when learning to perform it (e.g., Bingham et al., 2014; De Vries et al., 2015). Consequently, during the learning of a task, the anatomical independence that comes to characterize a mature action system may be viewed as the outcome of a process of increasing adaptation and refinement of the task. In this process the action system changes along with the task that requires its development. To make a start in tracing these changes the current study aims to determine to what extent the task for an action system in its initial development relies on environmental and anatomical components.

### Study Overview

To determine this in an experimental set up, we studied transfer effects. As transfer studies have shown the importance of the initial level of expertise on the extent of transfer (Barnett and Ceci, 2002; Rosalie and Muller, 2012) and as we are looking into the early development of an action system our aim required us to first of all include a pre-test that quantifies baseline performance (Rosalie and Muller, 2012). Second, the task that is to be learned should require a completely novel use of an anatomical part of the body; the participants should not be able to rely on previous experience in using their body in some way to perform the task. Third, the task should not only be novel to the participants, but should also be highly goal-directed (Rosalie and Muller, 2012; cf. Kelso and Zanone, 2002). In other words, it requires the development of a completely novel action system. Fourth, focusing on action systems in its initial stages of development implies that the transfer task should be “near” the training task in terms of the demands on performance and its temporal character (see Barnett and Ceci, 2002) – that is, to maximize chances of finding transfer effects, the design should aim to keep as much of the context of the training task unchanged and systematically vary only the aspects relevant to the research question (see Rosalie and Muller, 2012), given the processes of calibration and exploration, these are the goal and the anatomical aspects involved.

We devised an experiment that required participants to perform a computer game that was highly goal-directed and required modulating the electromyographical (EMG) signals of their arm muscles to perform. As a computer game, the task was highly goal-directed yet novel. Moreover, EMG current is typically a by-product of performing a task and is usually not a component part necessary to form a functioning action system. It thus introduces a new anatomical component to the task. Note, however, that learning to make use of such EMG current in a goal-directed way is not without application. For example, in rehabilitation, assistive technologies such as myoelectric prostheses require the development of action systems that embody these currents (see Smurr et al., 2008; Bouwsema et al., 2010; Pistohl et al., 2013).

As previous studies showed, exploration and calibration both help to differentiate activity as it is developing. Therefore, we do not expect a task for an emerging action system to be either fully defined relative to its goal or by the anatomy used. Rather, our main question in this study is: to what extent does the task for an action system in its initial development rely on environmental and anatomical components? We will answer this question by changing either the goal of the computer game or the musculature used to generate the EMG signals after a training period. If the task for the emerging action system is predominately anatomically defined, then transfer (i.e., the effect that the learning of one task has on the performance of a different task) occurs even if the goal of the task is changed across performances but musculature is kept the same. If an emerging action system is predominately goal-directed we expect that if the musculature used is changed, but the goal of the task is retained, then transfer will still occur.

To test these predictions, we used a pre-and post-test design. We had three experimental groups and a control group. As a pre- and post-test all groups performed a computer game in which the goal was to catch falling objects. In the test all groups used EMG of wrist muscles to control the game. As a training, the experimental groups had to perform a different game or used different musculature. First a group of participants learned to play the game with the same settings as during the testing condition. Since in the game objects needed to be caught with an effector that was controlled with wrist muscles, we call this condition “Catching-Wrist.” Second, we had a group that learned to play a computer game in which the goal of the game was to intercept falling objects (i.e., a different training game) – but the muscles used to control the game were the same (“Intercept-Wrist”). Third, we had a group that, like the Catching-Wrist group had the goal of catching objects, but used their upper arm muscles to do so (“Catching-Arm”). Fourth, we had a sham control group (“SHAM”) that played an unrelated video game.

When comparing the change in pre- to post-test performance groups, we expected that:

(i) If the task for an emerging action system is in part anatomically defined, then changing the goal but retaining the musculature used should enable transfer. Hence, we expect that the Intercept-Wrist group will then show significant improvement over the SHAM group from pre- to post-testing. Conversely, changing the musculature while retaining the goal should then reduce transfer. Hence, we expect that the Catching-Arm group will show significantly less transfer compared to the Catching-Wrist group.
(ii) If the task for an emerging action system is in part defined by the goal in the environment, then changing the musculature but retaining the goal should enable transfer. Hence in that case we expect the Catching-Arm group to show significant improvement over the SHAM group from pre- to post-testing. Conversely, changing the goal while retaining the musculature should then reduce transfer. Hence, we expect that the Intercept-Wrist group will show significantly less transfer compared to the Catching-Wrist-group.

## Materials and Methods

### Participants

Fifty-two able bodied adults participated (mean ± SD age: 21.90 ± 3.27 y); 13 men and 39 women. The participants (1) were all right handed, (2) had normal or corrected to normal vision, (3) were free of any (history of) disorders of the arms or upper body, and (4) had no prior experience in the use of myoelectric devices. The study was approved by the local ethics committee and an informed consent was obtained from all participants prior to the start of the experiment. Upon completion of the experiment all participants received a gift voucher.

### Materials

Two myogames were used – a Catching game and an Intercepting game – and both ran on a laptop computer. Two pairs of self-adhesive electrodes were connected to a desktop computer via a Porti-5 data acquisition device (TMS International, The Netherlands) that sampled the data at 500 Hz. Custom LabView software (National Instruments Corporation, USA) digitally rectified and filtered the signals (high pass filter, cutoff frequency 10 Hz; low pass filter, cutoff frequency 20 Hz) and fed the EMG signals from the electrodes to the laptop via UDP at 125 Hz. The games resampled the EMG signal at 50 Hz and logged all changes on the screen during play to a text file.

The SHAM control group trained a platform game called “Super Mario Bros,” which was run on a Nintendo Entertainment System (Nintendo Co. Ltd, Japan). This game was connected to a standard 32 cm (CRT) TV monitor.

### Myogames

#### Catching Game

**FIGURE 1:**
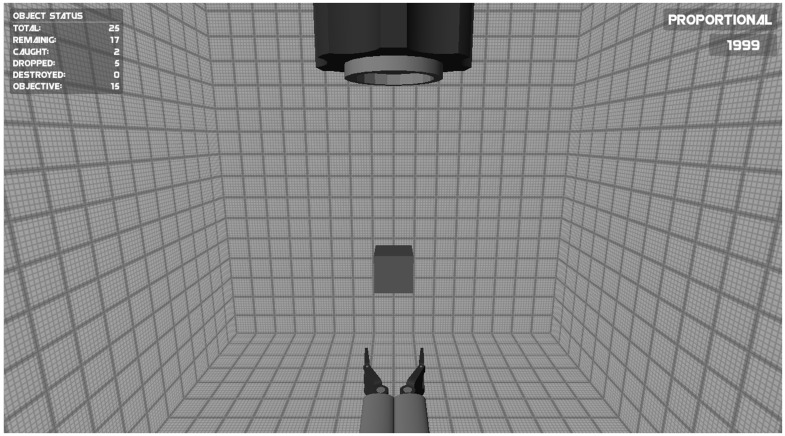
**Screenshot of the Catching game.** The opening and closing of the effector at the bottom of the screen was controlled using two myosignals (of the wrist muscles or of the upper arm muscles). The goal of the game was to catch falling objects with a effector so that the objects did not hit the ground (see text for details).

In the Catching game the objective was to catch falling objects with an effector so that the objects did not hit the ground. A screenshot of the game is shown in **Figure 1**. The falling objects had different shapes, each having a different color (light blue, blue, and red). The objects were given a random size (that never exceeded the maximum aperture of the effector). The objects that needed to be caught fell straight down from a “barrel” at the upper center of the screen. The effector used to catch the objects remained stationary at the bottom center of the screen. In order to catch the falling objects, the closing and opening movement of the effector (i.e., its aperture) was controlled using two myoelectric signals. The speed of the change in aperture of the effector was proportional to the amplitude of the EMG signals. To make sure that the game required accurate use of the EMG signal, two constraints were imposed on goal attainment. First, the aperture of the effector needed to be adapted to the size of the falling objects. If the aperture exceeded the diameter of the falling object more than 1.7 times, the effector started to vibrate and gave off “sparks” (shown in **Figure 1**). Subsequently exceeding the diameter of the object by more than 2.3 times would cause the effector to force closing rapidly. Second, the three shapes and colors of the falling objects represented their fragility (light, medium, strong). In this game the speed of closing the effector therefore needed to be adapted to the fragility of the object. If the virtual force exerted on the object reflected by the closing speed of the effector exceeded the object’s threshold, the object would break.

#### Intercepting Game

**FIGURE 2:**
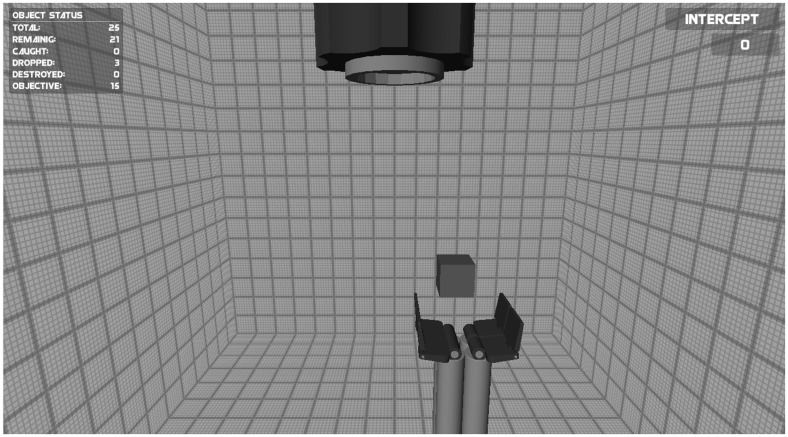
**Screenshot of the Intercepting game.** The speed of the effector at the bottom of the screen to the left and right was controlled using the myosignals (of the wrist muscles). The goal of the game was to intercept falling objects with the effector so that the objects did not hit the ground (see text for details).

The objective of the Intercepting game was to intercept falling objects with an effector so that the objects did not hit the ground. The game was identical to the Catching game group except (i) the aperture of the effector was fixed throughout the game, (ii) the objects could not break, and (iii) the objects that needed to be caught fell downward from a “barrel” at the upper center of the screen in any random direction (**Figure 2**). In this game not the aperture of the effector, but the effector’s movements to the left and right were controlled using the myoelectric signals. To make sure that the game required a high accuracy in using the myosignals the effector had large vertical edges (**Figure 2**). This ensured that the objects could only be intercepted by timing the positioning of the effector carefully. If the object made contact with the effector’s edges, the object would bounce away and the goal of intercepting it would not be obtained. The displacement speed of the effector was proportional to the amplitude of the EMG signals.

#### SHAM Game

The SHAM group, training in playing Super Mario Bros, had to control an avatar and safely guide the avatar through a world by jumping platforms and avoid enemies. The game was played using a standard hand held Nintendo controller, no control of a myosignal was implemented.

### Design

The experiment was conducted over the course of 4 days and consisted of a pre-test, three training sessions and a post-test. All groups performed the pre- and post-test, which consisted of playing one level of the Catching game using the EMG of the wrist muscles to control the game. On the first day, the pre-test was performed, which was followed by the first training session. On the second and third day the remaining two training sessions were conducted. To limit short-term interference effects from the third training session, the participants performed the post-test on the fourth day. Participants were randomly assigned to either the Catching-Wrist group (*n* = 13, 2 men, 11 women), the Intercept-Wrist group (*n* = 13, 4 men, 9 women), the Catching-Arm group (*n* = 13, 3 men, 10 women), or to the SHAM group (*n* = 13, 4 men, 9 women).

### Experimental Groups

#### Catching-Wrist Group

The Catching-Wrist group practiced playing the Catching game. They used the myosignals from the flexor and extensor muscles of the wrist. The signal from the flexor muscles acted to close the effector and the signal from the extensor muscles acted to open the effector.

#### Intercept-Wrist Group

The Intercept-Wrist group was identical to the Catching-Wrist group in all respects but one: in this group the Intercepting game rather than the Catching game was practiced. The goal of this game was to intercept falling objects. Activation of the flexor muscles moved the effector leftward whereas activation of the extensors moved the effector rightward.

#### Catching-Arm Group

The Catching-Arm group differed from the Catching-Wrist group only with respect to the musculature used to play the game. In the Catching-Arm group the game was not practiced using the wrist muscles, but by using the muscles of the upper arm. The signal from the lower part of the biceps muscle acted to close the effector and the signal from the lateral head of the triceps muscles acted to open the effector.

#### SHAM Group

The SHAM group practiced playing Super Mario Bros. The game was played using a standard Nintendo controller held in the palm of the hand and required no myosignal use.

### Procedure

#### Fitting of the Electrodes

Prior to playing one of the myogames, the electrodes were fitted by palpating for the most prominent muscle bellies of either the extensors and flexors of the wrist (for the Catching-Wrist and Intercept-Wrist) or the upper arm’s biceps or triceps muscle (Catching-Arm group) during contraction. The self-adhesive electrodes were subsequently placed at those sites. For the flexors and extensors of the wrist, the electrodes were placed above the proximal belly of the wrist and finger flexors (i.e., flexor carpi ulnaris, flexor digitorum superficialis, or profundus) and above the proximal belly of the finger and wrist extensors (i.e., brachioradialis, extensor carpi radialis longus, and brevis), respectively. For the flexor and extensor of the upper arm, the most distal (combined) part of the biceps muscle and the lateral head of the triceps muscle were used.

To ensure the placement of the electrodes remained constant within each participant throughout the experiment, the location of the electrodes was marked with a pen, both in the testing and the training sessions. The signals were digitally processed and sent to the game computer. In the game environment both signals were calibrated by determining the minimum and maximum value of each electrode independently and scaling each signal to a standard range before the game began. The signal was scaled and amplified so that the minimum and maximum speed of the effector conformed to 5 and 25%. The fitting procedure was repeated each day for each individual participant before training started.

#### Pre-test and Post-test

The pre-test was equal to the post-test. Participants were asked to play the first level of the Catching game, using the flexor and extensor muscles of the wrist. In this single testing level (level 1) 25 objects fell down and needed to be caught by controlling the effector. The level started when the experimenter pressed start and finished when the last object was caught or had fallen down. The participants received verbal instructions explaining the goal of the game – i.e., to try to catch the objects before they hit the ground – and how to control the effector.

#### Training Sessions

In each session all myogaming groups trained by playing their game for 20 min. Each game consisted of three levels that only differed (1) in the amount of objects to be caught before advancing to the next level (15 for level 1, 20 for level 2, and 24 for level 3, respectively) and (2) in the speed with which the objects fell down. At higher levels, more objects needed to be caught and the objects fell at greater speeds. The participants received concurrent feedback during their performance. They could, for example, monitor the number of objects that needed to be caught to advance to the next level, the current number of objects caught or missed and the number of objects that still remained. They also received feedback on the number of points scored (with each object caught). Upon finishing a level, a summary of these results was presented and, depending on the number of objects caught, the player would then either advance to the next level or play the same level again. After playing all three levels, the participants started again at level 1. The games had no sound.

The SHAM group played Super Mario Bros for 20 min per session. The participants only played the first four levels of the game (i.e., level 1–1 to 1–4) and then started over. The game was muted so that it had no sound.

### Data Analysis

All dependent variables used to determine in-game performance were calculated from the output file provided by the myogames using customized Matlab (The Mathworks Inc., USA) scripts. As playing the games proficiently required a high degree of accuracy in catching the objects, we looked primarily at accuracy to assess in-game learning effects. The accuracy was determined as the number of objects caught divided by the total number of objects that dropped from the “barrel.”

In order to scrutinize on performance, we explored several other aspects of performance. Accuracy is primarily determined by three aspects: (1) making sure not to open the effector too widely as this would cause it to force-close and miss the object. Therefore, we looked at the participant’s ability to adjust the size of the effector’s aperture to the size of the falling objects. We calculated this relative maximum aperture (RMA) as the maximum aperture of the effector per catch divided by the width of the falling object. Note that the RMA has an upper limit of 2.3, as opening the effector further would result in forced closing. (2) Making sure not to close the effector too far when catching as this would cause the objects to break. We therefore determined the mean peak EMG opening and closing signal from the 25 catches during the pre-test and post-test. (3) Making sure to close the effector at the right moment, otherwise the falling objects would either bounce off the effector or fall through. Therefore, we looked at the timing of the catch. We calculated the distance of the falling object to the effector at the moment that the peak EMG closing signal was generated for all catches in the pre- and post-test and analyzed their mean value and their variability (standard deviation) within the testing trial.

To determine changes in performance during learning, a repeated measures ANOVA was performed on the accuracy with Session (sessions 1, 2, and 3) as within subjects factor and Group (Catching-Wrist, Catching-Arm, Intercept-Wrist) as between subjects factor. *Post hoc* comparisons of the in-game performance were corrected for multiple comparisons using a Bonferroni correction.

To check for differences in trials performed between groups in the training, we performed a one-way ANOVA with group (Catching-Wrist, Catching-Arm, and Interception-Wrist) as the between subject factor. To check whether the groups differed in the number of trials performed we performed a Chi-square analyses.

In order to determine transfer effects, the change in performance was calculated from pre-test to post-test and this change was compared across groups. Before this comparison, we first performed a univariate ANOVA to check for initial differences between groups in pre-test performance. If this test would yield any differences between groups, the pre-test value would be added to the subsequent analysis as a covariate – there were, however, no pre-test differences in any of the dependent variables. Transfer effects for each of the above defined outcome measures was then determined by conducting an ANOVA on the change in performance with Group (Catching-Wrist, Catching-Arm, Intercept-Wrist, SHAM) as a between subjects factor. Effect sizes were calculated using generalized eta-squared ($\eta_{G}^{2}$ine-formula>; Olejnik and Algina, 2003; Bakeman, 2005).

Based on the change in accuracy, we set up specific hypotheses for each dependent variable beforehand. To test the first of our main hypothesis of whether the task was constituted by the anatomy involved, in a planned contrast on the change in accuracy (i) the Intercept-Wrist group was compared to the SHAM group and (ii) the Catching-Wrist was compared to the Catching-Arm. Likewise, to test the second main hypothesis of whether the task was constituted by the goal of the task (i) the Catching-Arm group was compared to the SHAM group and (ii) the Catching-Wrist was compared to the Intercept-Wrist.

Any improvement in accuracy may in part be the result of scaling the effector’s aperture to the size of the object. From our earlier experience with this task (Van Dijk et al., 2015) we know that novices in the test task open the effector too far, leading to low accuracy scores. Therefore we expected (i) the RMA of the Catching-Wrist group to have decreased from pre- to post-test significantly more than the SHAM group. As adjusting the aperture of the effector cannot be learned in the Intercepting game it was expected (ii) that the RMA for the Intercept-Wrist group would decrease significantly less than the Catching-Wrist group, while (iii) the Catching-Arm group is expected to decrease its RMA more than the SHAM group.

As generating large bursts of activation could result in either opening the effector too widely or in breaking the object that needed to be caught, we expected a decrease in peak EMG signal both for opening and closing the effector from pre-test to post-test. We expected (i) the Catching-Wrist group to have decreased its peak EMG signals from pre- to post-test significantly more than the SHAM group. Furthermore, it was expected (ii) that the Intercept-Wrist group would improve significantly over the SHAM group, and (iii) the Catching-Arm group would improve significantly over the SHAM group.

With respect to both the timing of the grasp and the variability in timing of the grasp, we expect the same patterns of results as in the RMA: (i) we expected that the Catching-Wrist group would improve performance significantly over the SHAM group. As the Intercept-Wrist group would to be unable to learn about the appropriate timing because the task-dynamics were unavailable in their training game (intercepting), we therefore, (ii) expected the Intercept-Wrist group to be significantly worse than the Catching-Wrist group. As the Catching-Arm group equally had experience in timing the grasp, (iii) we expected the Catching-Arm group to significantly improve over the SHAM group.

All these hypotheses were tested with planned comparisons (contrasts) in the ANOVA. For our two main hypotheses concerning the change in accuracy, we used two ANOVA’s with a different set of planned comparisons each. This meant that we had a total of four comparisons for the change in accuracy. For each of the two sets of hypotheses we therefore used a significance level of α = 0.025. All other analyses used a significance level of α = 0.05.

## Results and Discussion

### Participants

More females than males were participating in each of the experimental groups. Chi-square analyses did not show a relation between the groups and the sex distribution.

### Training Performance

**FIGURE 3:**
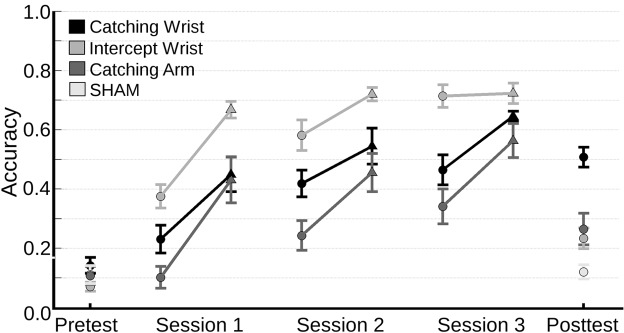
**Mean accuracy (and standard error of the mean) on the pre-test and post-test for all groups and on the three training sessions for all experimental groups.** Each point denotes the mean accuracy per trial (a trial consisted of 25 catches). To characterize the learning process across sessions the mean accuracy on the first trial playing level 1 (circles) and the last trial playing level 1 (triangles) for each 20 min session are shown.

Before looking into transfer effects the change of in-game accuracy was analyzed in order to ensure that the participants improved their performance as they learned. The in-game accuracy of all experimental groups across all sessions, and the accuracy of the SHAM group on the pre- and post-test is shown in **Figure 3**.

Visual inspection of the data showed that all groups increased their performance across sessions. Because participants advanced to higher levels of the games on the basis of their individual performances, and because the levels differed in difficulty, accuracy could not be compared straightforwardly across levels. So, we limited our analyses to the performance of the first trial of each session. We examined whether the learning had affected the normality of the data. Therefore, we first performed a Kolmogorov–Smirnov test, which was not significant [*D*(117) = 0.08, *p* = 0.08], showing that the data were normally distributed. A repeated measures ANOVA was performed on the accuracy of the first trial of each training session with Session as within subjects factor and Group as between subjects factor. The analysis revealed a main effect for Session [*F*(2,72) = 44.41, *p* < 0.001, $\eta_{G}^{2}$ = 0.46] and for Group [*F*(2,36) = 19.92, *p* < 0.001, $\eta_{G}^{2}$ = 0.53]. There was no significant interaction effect. A *post hoc* analysis revealed that the Intercept-Wrist group was more accurate than both the Catching-Wrist group (*p* = 0.003) and the Catching-Arm group (*p* < 0.001) in playing their respective myogame. Moreover, the Catching-Wrist group, using their wrist muscles, was more accurate than the Catching-Arm group that used the muscles of the upper arm (*p* = 0.029).

To examine whether this difference in training performance between groups was visible in the number of trials performed, we tested whether the total number of training trials differed between the groups with a one-way ANOVA with group (Catching-Wrist, Catching-Arm, and Interception-Wrist) as the between subject factor. The total number of completed trials averaged over participants within a group is shown in **Table 1**. Each trial consisted of 25 catches or interceptions.

**Table 1:** Mean total number of trials performed during the practice sessions (and standard error of the mean) for all training groups and the total number of trials completed by all participants from each group together for each level (a trial consisted of 25 catches or interceptions).

		Total number of trials		
	Mean total number of trials	Level 1	Level 2	Level 3
Catching-Wrist	37.38 ± 0.68	236	221	23
Intercept-Wrist	52.31 ± 0.68	128	316	235
Catching-Arm	38.00 ± 0.52	295	161	29

*The total number of trials divided by the number of participants in each group does not exactly amount to the mean total number of trials because not every participant performed the same number of trials*.

We found that the Interception-Wrist group performed more trials than the other groups [*F*(2,38) = 179.54, *p* < 0.001, $\eta_{G}^{2}$ = 0.91]. We also checked whether there was a difference among groups with regard to the number of trials that were performed at each level. The total number of trials completed at each level by all participant within each group is shown in **Table 1**. As the objects fell faster at higher levels, it was expected that such an association occurred. Indeed, the analyses showed an association between the group and the number of trials played in each level (χ^2^(4) = 347.35, *p* < 0.001). Taken together, these results suggest that the Catching-Arm game was the hardest task to learn, while the Interception-Wrist game was comparatively easy to learn.

### Transfer Performance

To summarize our main results the mean difference from pre-test to post-test and the confidence interval of the mean of all dependent variables for each group is shown in **Table 2**.

**Table 2:** Mean change [and 95% confidence interval of the mean] in all dependent variables from pre- to post-test for all groups.

	Catching-Wrist	Intercept-Wrist	Catching-Arm	SHAM
Accuracy	0.37 [0.28;0.45]	0.16 [0.10;0.23]	0.16 [0.06;0.25]	0.01 [–0.09;0.11]
Relative maximum aperture (RMA)	-0.33 [-0.49;-0.18]	-0.21 [-0.34;-0.07]	-0.16 [-0.32;0.00]	-0.07 [-0.27;0.14]
Peak opening signal	-0.03 [-0.23;0.18]	0.00 [-0.18;0.19]	0.06 [-0.09;0.22]	0.02 [-0.14;0.19]
Peak closing signal	0.13 [-0.02;0.29]	0.13 [0.01;0.24]	0.13 [0.02;0.24]	0.09 [-0.02;0.19]
Timing of the grasp	-0.67 [-0.88;-0.46]	-0.29 [-0.56;-0.02]	-0.13 [-0.54;0.28]	-0.28 [-0.72;0.17]
Variability in timing of the grasp	-0.80 [-1.01;-0.60]	-0.13 [-.34;0.09]	-0.27 [-0.63;0.10]	0.02 [-0.24;0.28]

#### Accuracy

**FIGURE 4:**
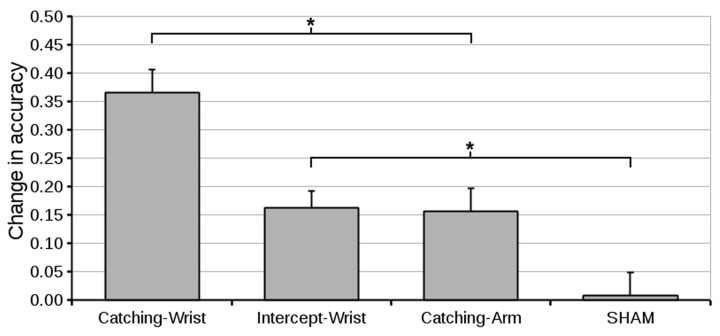
**Mean difference in accuracy (and standard error of the mean) from pre-test to post-test for all groups.** The Catching-Wrist group improved most while the SHAM group showed no improvement. The Catching-Arm and Intercept-Wrist groups both showed improvement in between the other two groups (^∗^ indicate significant differences in planned contrasts, see text for details).

To test our main hypotheses we compared the change in accuracy from pre-test to post-test of all the different groups. An ANOVA on the level of accuracy at the pre-test (**Figure 3**) with Group as a between subjects factor revealed no Group effects. The pre -to post-test difference is depicted in **Figure 4**. The accuracy in the Catching-Wrist group has increased the most while the SHAM group showed no improvement. The two other groups appear to show an increase in performance in between the Catching-Wrist and the SHAM group.

To see whether these differences hold statistically we looked into the change in performance from pre-test to post-test using an ANOVA on the pre- to post-test difference in accuracy, with Group as between subjects factor. The general analysis showed a main effect of Group [*F*(3,48) = 13.31, *p* < 0.001, $\eta_{G}^{2}$ = 0.45]. Following this analysis our main hypotheses were tested using planned contrasts comparing the group effects. Our first set of hypotheses tested whether the task was in part constituted by the anatomy involved. Our planned contrast showed that the Intercept-Wrist group improved significantly compared to the SHAM group (*p* = 0.009). Moreover, it showed that Catching-Wrist improved significantly compared to the Catching-Arm group (*p* = 0.001). Both these results indicate that transfer occurred if the anatomy was retained.

Our second set of hypotheses tested to what extent the task was constituted by the goal of the task. Our planned contrast showed that the Catching-Arm group improved significantly compared to the SHAM group (*p* = 0.012). Moreover, it showed that the Catching-Wrist improved significantly compared to the Intercept-Wrist group (*p* = 0.001). Both these results indicate that transfer also occurred if the goal of the task was retained.

#### Relative Maximum Aperture

**FIGURE 5:**
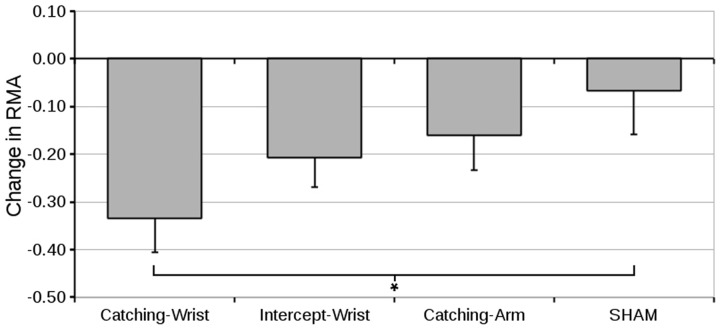
**Mean difference in relative maximum aperture (and standard error of the mean) from pre-test to post-test for all groups (^∗^indicate significant differences in planned contrasts, see text for details)**.

The pre- to post-test differences in the RMA are shown in **Figure 5**.

An ANOVA on the pre-test value showed no significant differences between groups (grand mean RMA was 1.57 ± 0.04). We therefore conducted an ANOVA on the pre- to post-test differences with Group as between subjects factor. The change in RMA from pre-test to post-test can be seen in **Figure 5**. There was no significant overall Group effect. Only the first pre-specified contrast was significant – that is, only the Catching-Wrist group differed significantly from the SHAM group (*p* = 0.015).

#### Peak EMG Signal

**FIGURE 6:**
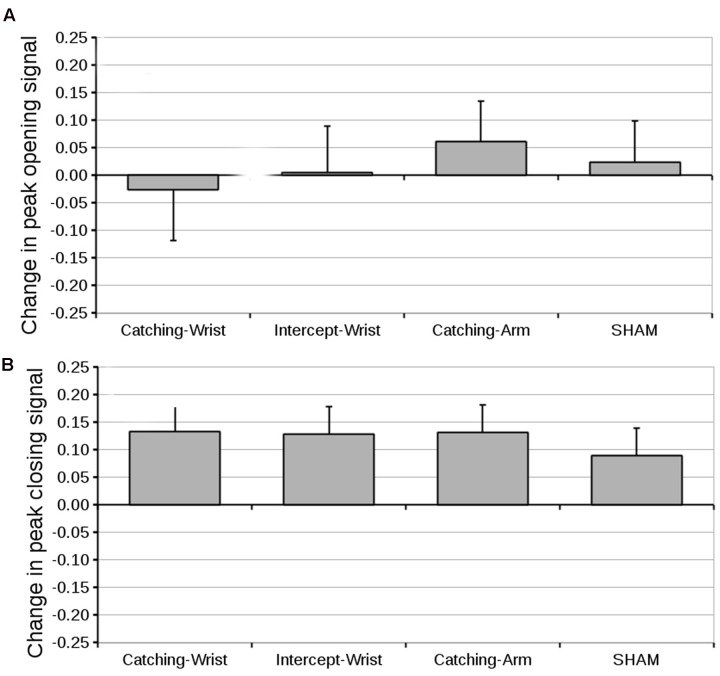
**(A)** Mean difference in peak opening signal (and standard error of the mean) from pre-test to post-test for all groups. **(B)** Mean difference in peak closing signal (and standard error of the mean) from pre-test to post-test for all groups.

The differences in peak opening and closing EMG signals are shown in **Figures 6A,B**, respectively. As ANOVA’s on the pre-test peak opening EMG and on the pre-test closing EMG revealed no differences between groups in initially generated peak EMG (grand mean opening signal, 0.72 ± 0.05, closing signal, 0.61 ± 0.03), we compared the pre- to post-test difference with Group as between subjects factor. There were no significant effects for Group either for the peak opening EMG or for the peak closing EMG. Planned contrast also showed no significant differences.

#### Timing of the Grasp

To examine to what extent the timing of the catch determined the improvement in accuracy we analyzed the distance of the falling object to the effector at the moment of the peak closing EMG signal (henceforth “start of the grasp”). The effector was located at position 0 (the objects started at position 3.8).

**FIGURE 7:**
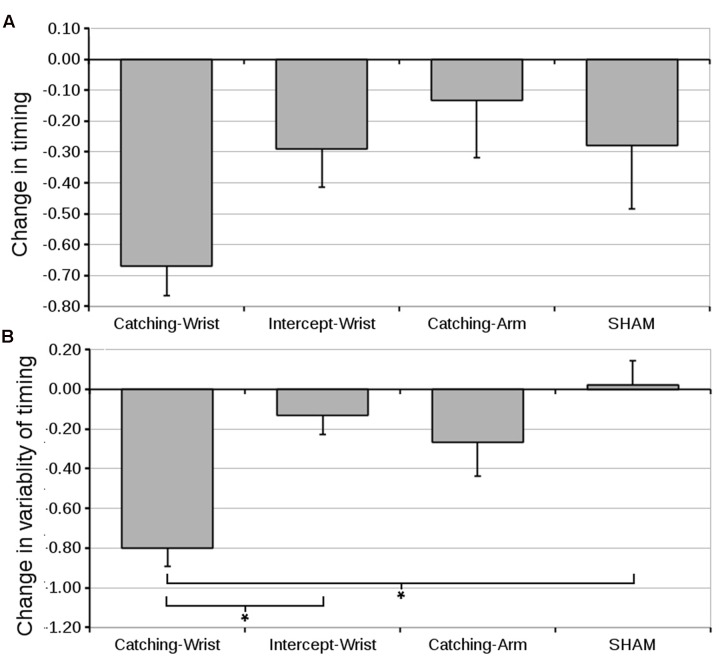
**(A)** Mean difference in distance of the object to the effector at the moment of peak closing signal (and standard error of the mean) from pre- to post-test for all groups. **(B)** Mean difference in standard deviation of the distance of the object to the effector at the moment of peak closing signal (and standard error of the mean) from pre- to post-test. Although the absolute timing of the grasp appeared not to have been critical (the object is catchable along a large trajectory) improvement in accuracy (**Figure 4**) is closely matched by a decrease in variability of the timing of the catch (^∗^ indicate significant differences in planned contrasts, see text for details).

An ANOVA on the start of the grasp on the pre-test revealed no significant differences between groups (grand mean 0.82 ± 0.09). The mean difference in the start of the grasp from pre-test to post-test is represented in **Figure 7A**. An ANOVA on the pre- to post-test difference showed there was no significant Group effect. None of the planned contrasts revealed a significant difference between groups.

However, depending on the size (length) of the object, the maximum aperture of the effector and the magnitude of the peak closing EMG signal, the absolute distance of the object to the effector may not be critical for the participant to catch the object. Indeed, judging from the standard error in **Figure 7A**, there is considerable variability between participants in absolute timing of the start of the grasp. Within a participant, the variability in timing the grasp may, however, still stabilize and thus help to improve performance. To characterize improvement in the timing of the catch, we therefore decided to look at the within subject variability in timing the closing signal by calculating the standard deviation of the start of the grasp across all 25 catches of the pre-test and post-test for each participant.

#### Variability in Timing the Closing Signal

An ANOVA on the mean standard deviation of the start of the grasp revealed no significant differences between groups (grand mean 1.29 ± 0.06). The difference in mean standard deviation of the start of the grasp from pre-test to post-test can be found in **Figure 7B**. An ANOVA on the pre- to post-test differences in variability in timing the start of the grasp with Group as between subjects factor revealed a significant effect for Group [*F*(3,52) = 8.46, *p* < 0.001, $\eta_{G}^{2}$ = 0.35]. As expected, the first planned contrast showed the Catching-Wrist decreased the variability in timing the grasp significantly over the SHAM group (*p* < 0.001). The second expectation was also confirmed: the Intercept-Wrist group was significantly more variable than the Catching-Wrist group (*p* < 0.001). The third planned comparison showed that the Catching-Arm group did not differ significantly from the SHAM group.

## General Discussion

In this study, we set out to determine to what extent the task for an action system in its initial development relies on environmental and anatomical components. Our main finding on the accuracy of performance indicates that retaining either the environmental goal of the task or the musculature used will equally increase performance relative to training a control task. However, in comparison to training the test, changing either the goal or the musculature will also equally decrease performance. These findings indicate that in the initial development of an action system, the task to which the system pertains is not specified solely by either the goal of the task or the anatomical structures involved. It is both the goal of the task as well as the anatomical structures involved that contribute to the initial formation of an action system for a task. This suggests that the anatomical independence that comes to characterize a fully formed action system for that task is the outcome of a learning process – as is any anatomical specificity that is required (see Bingham et al., 2014).

By scrutinizing on measures of performance at the level of the actions within a trial, we hoped to be able to find indications of either exploration of information or of calibration to information during the learning process. Looking at defining characteristics of the catching behavior in the test task, we were unable to find much systematic changes across learning. Neither the peak EMG signals generated nor the timing of the closing of the effector appeared to reflect changes in performance between groups. The relative maximum opening and the variability of the timing tended to change in the same direction as our main accuracy measure, but these trends too failed to reach significance. It seems that, in our task, the overall accuracy was the best characterization of task performance. This might not be surprising, because the objective, in terms of the instructions given to the participants, was to try and catch or intercept as many of the objects as possible. Therefore, it seems reasonable to assume that the action system forms at this level of performance.

An object for future study might be to try and flesh out the role of the processes of calibration and exploration during learning. This might require scrutinizing on the behavior within single participants. That is, recent evidence has shown that during learning there are large individual differences in the information used (Withagen and Van Wermeskerken, 2009; Dicks et al., 2010). Moreover, in several learning studies it has been shown that the learning toward the use of information differs between individuals (Jacobs and Michaels, 2007; Golenia et al., 2014; Vegter et al., 2014). These two individual differences across learning – both in the information the learner starts out with and in the trajectory subsequently followed – might cause different participants to have different sensitivities to changes in the task from training to the post-test. This might have resulted in variability of performance, and could have clouded systematic differences between groups on the post-test. Although we have explored for changes in behavior within participants, our analyses so far were not successful.

What might be the reason that we had not found clear indications of how task goal and anatomy interacted? Our results suggest that the anatomical independence that comes to characterize a mature action system can be viewed as the outcome of a process of increasing differentiation of the task over learning. In this process of both calibration (e.g., Bingham et al., 2014) and exploration (e.g., De Vries et al., 2015), the action system changes along with the task to which it pertains. That is, as achieving the task requires a more finely attuned action system, the system refines its coordination. Conversely, doing so allows the learner to perceive more and more subtle ways to perform the task. By emphasizing this reciprocal differentiation of *both* action and goal during the learning of a task (see Gibson and Gibson, 1955), there is no principled reason for not accounting for anatomical constraints within an action systems perspective. Anatomical constraints can come to define an action system in so far as they constrain goal attainment (see Bingham et al., 2014). The theory of action systems is thus rich enough to deal equally well with functional as well as anatomical specificity and might thus be applied much wider than it currently is.

One of the fields in which the action systems approach might contribute is that of motor recovery. Even though two of our experimental groups used a completely different set of muscles or the game consisted of a different kind of action, both improved significantly over controls in their ability to play the Catching-Wrist game. Both are innovative findings for motor recovery. For example, although seldom tested, in literature on EMG control the necessity of using similar musculature is often assumed (see Dupont and Morin, 1994; Dawson et al., 2011; but see Romkema et al., 2013). Our finding of task-specific transfer might thus be helpful in developing novel training programs for learning to use an EMG signal to handle prostheses and other EMG controlled assistive devices (see Bouwsema et al., 2010). Moreover, our finding of transfer in the absence of task-similarity is one of the first to provide empirical support for using muscle-specific EMG training in rehabilitation (e.g., Smurr et al., 2008; Pistohl et al., 2013; Terlaak et al., 2015). Combining these results with a differentiation account of learning, our current study suggests such a role primarily in the initial stage of learning (see also Van Dijk et al., 2015, 2016b).

The current experiment is, however, not without its limitations. First of all, because our participants advanced to higher levels of difficulty as their skills increased and because these levels were higher paced, we found that the group with the largest learning effect (in terms of in-game accuracy) also had the highest number of training trials within an equal training duration. We decided to control the training duration and control the intensity of training by having each participant advance to higher levels as their individual skills improve. The fact that the number of training trials did not affect transfer in our study supports this design choice. However, in future research it might be better to keep the duration of each trial across levels constant. Second, within our sample, females were overrepresented. To our knowledge there are no differences between males and females in their ability to transfer (myoelectric) skills, however, our experiment does not allow us to rule out such effects. Third, although none of our participants had any experience in using myoelectric devices, we have not controlled for any interception experience. Since intercepting moving objects is common in many sports and leisure activities we assume such expertise will be similar across groups. However, note that Terlaak et al. (2015) did not find a relation between simple motor control tasks and myoelectric control skills.

An important collateral of the action system approach is that it takes action to be the basic component and views anatomy as a derived classification (Reed, 1988; see Van Dijk et al., 2016b). That is, in this view, it is only in the context of acting that anatomical properties can be distinguished as relevant. This fits for example with the interpretation that Bingham et al. (2014) gave of their results when they suggested that the “relevant anatomical properties must be incorporated into the functional dynamics of calibration” (p. 68). That is, what counts as *relevant* anatomy, is determined in learning to adapt to the task. We add to this the converse idea, that equally, what counts as the goal of the task is, in part, differentiated by the anatomy available (see De Vries et al., 2015). Over learning both anatomical and environmental aspects form in the context of the task that is differentiating as the participant acts (Newell, 1986).

Taking this point one step further, this interpretation can also have an important consequence for our understanding of transfer. In our study we used transfer, i.e., the effect of past performance of one task on the subsequent performance of another task, to establish a prior similarity between tasks. For example, finding transfer from the Catching-Wrist to the Catching-Arm group is then interpreted as showing that the tasks in both cases already share a similarity in goals, and therefore transfer occurred.

This is a common assumption: similarity in performance across tasks is often explained by assuming similarity in “identical elements” (Woodworth and Thorndike, 1901; see Rosalie and Muller, 2012), or similarity in “abstract contextual cues” (Rosalie and Muller, 2012). Despite research having shown a variety of interacting dimensions along which tasks might be said to share more or less similarity (Barnett and Ceci, 2002), the assumption that such similarity is an underlying precondition remains. In fact, even theories that relate (in)directly to Reed’s theory of action systems, often seek to explain similarity in performance by appealing to the learning of a pre-existing similarity of a more abstract and general kind. For example, the (representations of) coordination dynamics that were used to characterize performances over time, easily turns into the source of the learning process (see Kelso and Zanone, 2002, p. 795 pointing to this) and by proposing learning to attune to information is guided by information for learning, a more abstract similarity across tasks is introduced (Jacobs and Michaels, 2007, p. 336). Against the background of the foregoing discussion, this assumption that similarity across performances can only be accounted for by prior similarity of the parts that make up the performing system can now be questioned (see Shotter, 1983; Newell, 1996; Kelso and Zanone, 2002). Just as anatomical and functional relevance can be understood as two emerging aspects of learning a task, more generally, finding transfer between performances can also be taken to show that the participant was able to *achieve* similarity across performances in acting.

Although our results show that different aspects that persist across tasks enhance transfer, they moreover suggest that tasks are equally held together over time, by a differentiating learner. When learning a skill, one might not need to learn about prior anatomical or goal-relevant similarities of the environment, nor about similarity of a more abstract kind; one merely needs to become selectively receptive to the changing possibilities for action (Michaels and Carello, 1981; Reed, 1996; Rietveld and Kiverstein, 2014). Responsiveness in one task, say of rock climbing, might then cultivate a receptivity to action possibilities that allows for a continuity of performance into another task, namely that of ice-climbing (see Seifert et al., 2013).^1^

Selective receptivity to the ongoing possibilities for action might be enough to achieve continuity in performances over time. Continuity that can then be characterized through coordination dynamics or information space, without assuming these abstractions in turn underlie the learning process. It allows for *continuity* in the organism-environment *relation* over time without assuming prior similarity in any of its parts. Similarity, we suggest, is not the source of an action system and a task co-evolving, but it is the continuous outcome of this process.

## Ethics Statement

This study was approved by the Ethics Committee for Human Movement Sciences, University of Groningen, the Netherlands. All participants received written and oral information concerning the nature of the experiment. After that a written and signed informed consent was obtained from all participants.

## Author Contributions

LD contributed to the conception, design, data acquisition and analysis, and interpretation of the work. AH contributed to the data acquisition and analysis, and interpretation of the work. CS and RB contributed to the conception, design, data analysis, and interpretation of the work.

## Conflict of Interest Statement

The authors declare that the research was conducted in the absence of any commercial or financial relationships that could be construed as a potential conflict of interest. The reviewer HB declared a past co-authorship with two of the authors RB and CS to the handling Editor, who ensured that the process met the standards of a fair and objective review.

## Footnotes

1 Within cognitive psychology, such an approach has also been forwarded by Healy et al. (2005, 2006). Akin to learning an action system, this framework emphasizes the “procedural” specificity of transfer. By learning task-specific “inhibitions” here too the selectivity of an organism responding to the possibilities in the world is emphasized (Healy et al., 2006).
